# Natural-Course Evaluation of Infants with Positional Severe Plagiocephaly Using a Three-Dimensional Scanner in Japan: Comparison with Those Who Received Cranial Helmet Therapy

**DOI:** 10.3390/jcm10163531

**Published:** 2021-08-11

**Authors:** Takanori Noto, Nobuhiko Nagano, Risa Kato, Shin Hashimoto, Katsuya Saito, Hiroshi Miyabayashi, Mari Sasano, Koichiro Sumi, Atsuo Yoshino, Ichiro Morioka

**Affiliations:** 1 Department of Pediatrics and Child Health, Nihon University School of Medicine, Tokyo 173-8610, Japan; noto.takanori@nihon-u.ac.jp (T.N.); nagano.nobuhiko@nihon-u.ac.jp (N.N.); kato.risa@nihon-u.ac.jp (R.K.); 2 Noto Children’s Clinic, Tokyo 179-0084, Japan; 3 Kasukabe Medical Center, Department of Pediatrics, Kasukabe 344-8588, Japan; fight.together.0119@gmail.com (S.H.); katsuya-saito@nifty.com (K.S.); miyabayashi@dr.memail.jp (H.M.); 4 Department of Neurological Surgery, Nihon University School of Medicine, Tokyo 173-8610, Japan; sasano.mari@nihon-u.ac.jp (M.S.); sumi.koichiro@nihon-u.ac.jp (K.S.); yoshino.atsuo@nihon-u.ac.jp (A.Y.)

**Keywords:** cranial asymmetry, improvement, natural course, severity, scanner

## Abstract

This study aimed to clarify the natural course of positional plagiocephaly using a three-dimensional (3D) scanner and investigate the effectiveness of cranial helmet therapy (CHT). One hundred infants with severe plagiocephaly who visited our institutions between April 2020 and March 2021 were included. Cranial shape was measured using an Artec Eva 3D scanner. A cranial asymmetry (CA) >12 mm was diagnosed as severe plagiocephaly. An infant whose CA subsided to <12 mm was considered to have improved naturally or by CHT. The difference in CA between the second and initial scans was defined as the improvement value (median scan interval was two months). In the natural-course group comprising 56 infants with severe plagiocephaly, 37 (66%) with a median CA of 15.6 mm exhibited no improvement after two months. In the scan age- and evaluation interval-matched case-control study, the CA value in the CHT group improved by three times that in the natural-course group (−4.6 mm [*n* = 33] vs. −1.55 mm [*n* = 24], *p* < 0.001). Severe plagiocephaly did not improve naturally in 66% of the cases. Therefore, CHT should be considered if the CA is >12 mm on the initial evaluation.

## 1. Introduction

Positional plagiocephaly is the most common type of cranial asymmetry in infants. The prevalence of plagiocephaly is age-dependent; it was 16.0%, 19.7%, 9.2%, 6.8%, and 3.3% at 6 weeks and 4, 8, 12, and 24 months of age, respectively [1]. In Japan, as infants are traditionally laid on their backs, plagiocephaly has commonly been observed and culturally accepted. Many pediatricians and gynecologists have generally provided assurance that the infants’ cranial shape would improve naturally [2,3]. The parents are usually instructed to observe the infants on medical checkups at 3–4 months or 6–7 months of age because plagiocephaly improves naturally. Many infants with plagiocephaly have been confirmed during medical checkups at 9–10 months and 1.5 years of age in Japan. At the time of visiting the cranial-deformity outpatient department, therefore, the initiation of cranial helmet therapy (CHT) to correct the cranial shape is usually overdue for many infants, which is a current problem in clinical practice in Japan.

Although the total prevalence of plagiocephaly has been previously reported [1,4], no studies have examined the clinical characteristics according to severity. The severity of plagiocephaly is assessed using cranial asymmetry (CA, mm) [5,6,7,8]. Regarding the definition of plagiocephaly, there is no professional consensus on the optimal method of subjectively or objectively classifying plagiocephaly severity. In this study, based on previous reports, the severity of plagiocephaly was defined as follows: mild, CA 3–10 mm; moderate, 10–12 mm; and severe, ≥12 mm [5,6,7]. Another report has defined mild-to-moderate as 3–12 mm [8]. In either case, however, a CA of ≥12 mm was defined as “severe.” We hypothesized that many severe plagiocephaly with CA > 12 mm would not improve naturally. Since CA measurements can be objectively evaluated using a three-dimensional (3D) scanner [9,10,11], the first objective of this study was to clarify the natural history of severe plagiocephaly with CA > 12 mm by objective evaluation using a 3D scanner (Study 1).

Repositioning and physiotherapy are the optimal treatment choice for patients younger than four months of age who have mild or moderately severe plagiocephaly. Combined treatment with CHT, repositioning, and physiotherapy are the most beneficial management of infants older than four months of age with severe plagiocephaly or with worsening of mild or moderate plagiocephaly trialed on repositioning and physiotherapy [6]. Infants with severe plagiocephaly should be considered for CHT at any age [6], especially at 4–8 months of age [2,8,12,13,14].

Because many published reports proved the efficacy of CHT in the treatment of plagiocephaly, CHT initiation is recommended within 4–8 months [2,8,12,13,14]. In Japan, CHT for infants with plagiocephaly was initiated by Aihara in 2007 [2]; however, it remains an uncommon practice in Japan, unlike in other countries worldwide. Two studies in Japan have provided evidence for CHT, and they were single-arm, non-randomized studies that did not include untreated infants as a control [2,3]. Therefore, most parents have not been provided with evidence-based information of the cares and treatments of their infants. Since the recommended starting age for CHT is 4–8 months, parents do not have sufficient time to make an informed decision regarding CHT according to the severity of CA. Due to a lack of evidence-based information, it is possible that many parents in Japan are reluctant to use CHT, even in the cases of severe plagiocephaly with CA > 12 mm. Therefore, it is necessary to demonstrate CHT’s effectiveness in improving CA in plagiocephaly to the parents. In addition, no scientific studies examining plagiocephaly have comparatively analyzed the natural-course and CHT outcomes in infants of similar severity over the same evaluation period. In the second study, we initially evaluated infants with severe plagiocephaly with or without CHT twice using a 3D scanner, which made it possible to match the severity and evaluation age. The second objective of our study was to compare the improvement in severe plagiocephaly between infants with and without CHT to determine the efficacy of CHT (Study 2).

## 2. Materials and Methods

### 2.1. Subjects and Study Design

Two studies were performed. In Study 1, we examined the transition of severe plagiocephaly during the natural course. In Study 2, we examined the effect of CHT on severe plagiocephaly compared to that of the natural course. The study subjects comprised healthy infants who attended three medical centers in Japan between 1 April 2020, and 31 March 2021, for cranial deformities. Three-dimensional scanners had not yet been approved as medical devices by the Pharmaceuticals and Medical Devices Agency in Japan. Written informed consent was obtained from the parents or guardians of all the participants. The scanner evaluation interval was usually set to 2 months so that CHT could be started if the patient’s condition deteriorated. This study was approved by the Ethics Committees of the participating institutions (Kasukabe Medical Center and Noto Children’s Clinic: approval number 2019-032 and Nihon University Itabashi Hospital: approval number RK-200512-2).

**Figure 1:**
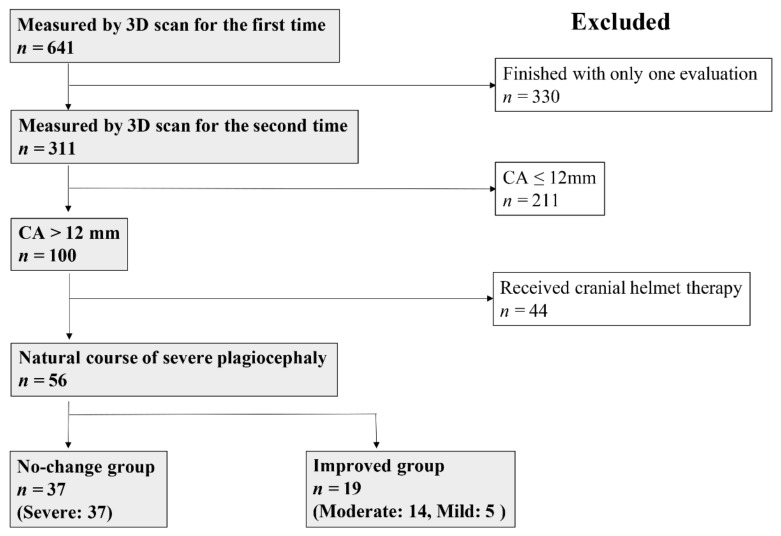
Flowchart of the enrolled infants with severe plagiocephaly following the natural course for Study 1. CA, cranial asymmetry; 3D, three-dimensional.

Study 1: Infants with plagiocephaly were included in a prospective, non-randomized, longitudinal observational study (Figure 1). Of the 641 infants who underwent 3D scan measurements, 311 participated with the second measurement (The remaining 330 rejected the second measurement). One hundred infants with CA > 12 mm, i.e., severe plagiocephaly, at the time of initial evaluation were enrolled. Because 44 infants who received CHT were excluded, 56 were included in the natural-course group. In the second evaluation, the infants were classified into the no-change group if the severity of plagiocephaly remained severe (*n* = 37) and improved group if the severity subsided to moderate or mild (*n* = 19).

**Figure 2:**
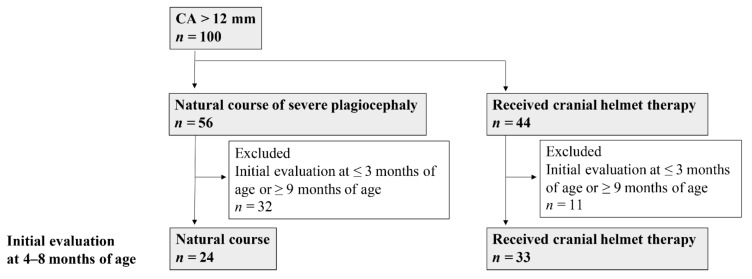
Flowchart of the enrolled infants with severe plagiocephaly in the natural-course and CHT groups for Study 2. CA, cranial asymmetry.

Study 2: Infants with severe plagiocephaly were included in a scan age- and evaluation interval-matched case-control study (Figure 2). Of the 100 infants with CA > 12 mm, i.e., severe plagiocephaly, 56 infants were included in the natural-course group and 44 in the CHT group. To match their ages at evaluation, only cases with an initial evaluation age of 4–8 months were selected (24 infants in the natural-course group and 33 in the CHT group) because CHT initiation is recommended at 4–8 months of age [2,6,12,13,14].

### 2.2. Study Methods

Study 1: Obstetric factors (birth weight, birth order, sex, gestational age, delivery mode, fetal presentation, and nutrition), initial evaluation age, second evaluation age, evaluation interval, CA, Cranial Vault Asymmetry Index (CVAI), their improvement value, head circumference, and growth of head circumference were investigated in the 56 infants with severe plagiocephaly following the natural course and subsequently compared between the no-change and improved groups. CA was used as an index for classifying the no-change or improved group to determine whether the severity of plagiocephaly had improved in the second evaluation (Figure 1).

Study 2: Obstetric factors, initial evaluation age, second evaluation age, evaluation interval, CA, CVAI, their improvement value, head circumference, and growth of head circumference were compared between the natural-course and CHT groups. The percentage of infants whose plagiocephaly severity improved from severe to moderate or mild was compared. In addition, the degree to which CA values improved in the natural-course and CHT groups was calculated.

### 2.3. Primary Outcomes

In Study 1, the primary outcome was the percentage of severe plagiocephaly cases in the second evaluation. In Study 2, the primary outcomes were the percentage of severe plagiocephaly cases in the second evaluation with or without CHT and the change in the improvement value of CA in infants with or without CHT.

### 2.4. Definition of Severity and Improvement

The severity of CA was defined as mild (3–10 mm), moderate (10–12 mm), or severe (>12 mm) [5,6]. An infant with CA > 12 mm was diagnosed with severe plagiocephaly [5,6]; therefore, all the infants included in this study had CA > 12 mm. Improvement was defined as a transition to “moderate or mild” from “severe”. Improvement in CA value was calculated as follows: second evaluation CA-initial evaluation CA. Improvement in CVAI value was calculated as follows: second evaluation CVAI-initial evaluation CVAI (See Section 2.6). Growth of head circumference was calculated as follows: second evaluation of head circumference–initial evaluation of head circumference.

### 2.5. Data Acquisition Using the 3D Scanner

A complete 360° scan of the cranial shape, including both ears, was performed using the Artec Eva 3D scanner (Artec, Inc., Luxembourg, Luxembourg). The head was protected using an elastic wig cap to prevent hair obstruction. The 3D resolution was 0.2 mm and accuracy 0.1 mm.

### 2.6. Data analysis Method

**Figure 3:**
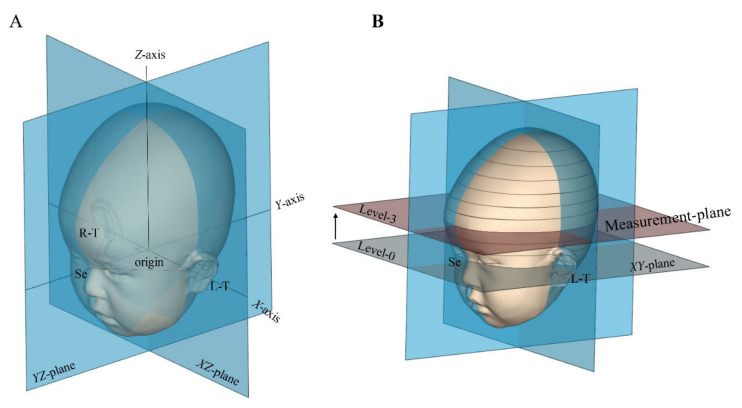
Three-dimensional images. (**A**): The methods by which the XY plane, X-axis, Y-axis, and Z-axis were determined. (**B**): The methods by which the measurement plane was determined. L-T, left tragion; R-T, right tragion; Se, sellion.

The data obtained were analyzed using Artec Studio image analysis software (Artec, Inc., Luxembourg, Luxembourg) to obtain 3D images and determine cranial shape. To align the 3D datasets in the virtual space, a coordinate system was established based on three anatomically defined reference points (left tragion, right tragion, and sellion). The aligning procedure has been performed in a previous study [2,9,11]. Figure 3A shows the methods by which the reference plane, X-axis, Y-axis, and Z-axis were determined. The sellion was set at the most concave point in the soft tissue at the nasofrontal angle between the forehead slope and the proximal nasal bridge. The tragion was set at the upper margin of the tragus. After setting these landmarks, the origin was set at the midpoint between the right and left tragions. Thereafter, the Y-axis was defined as a line through the sellion and the origin. The X-axis was defined as the line perpendicular to the Y-axis that crosses the origin. The XY plane was defined from the X- and Y-axes passing through the origin. The Z-axis was defined as the line perpendicular to the XY plane that crosses the origin. Figure 3B shows the methods used for the measurement plane. The XY plane was the reference cross-sectional plane (level 0). The portion of the cranium superior to the reference plane was divided into 10 equally spaced cross-sectional planes, each parallel to the reference plane, where level 10 was the plane through the vertex. Anthropometric measurements on level 3 planes were used in this study.

**Figure 4:**
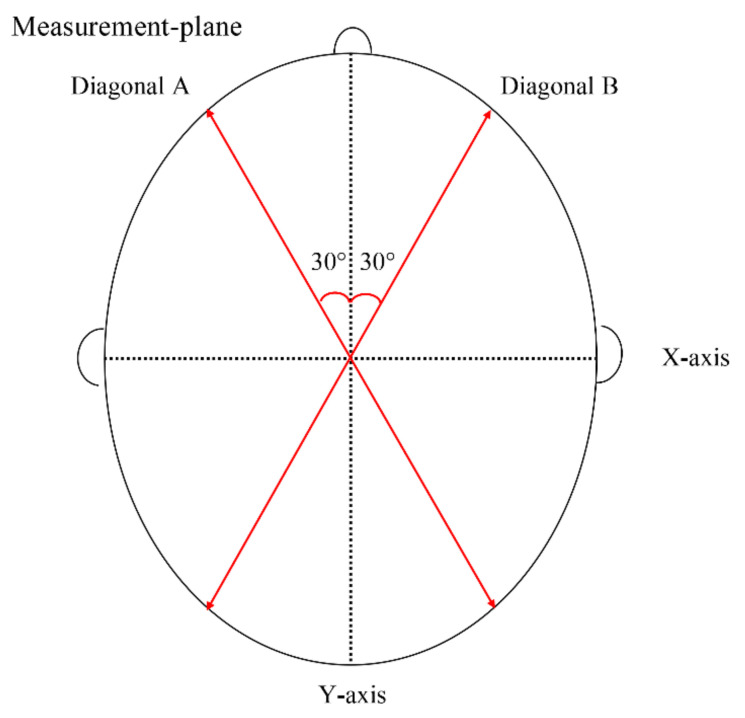
Measurement method for cranial asymmetry and cranial vault asymmetry index. CA (mm) = Diagonal A − Diagonal B. CVAI (%) = CA/Diagonal B × 100% (where diagonal A is greater than B). CA, cranial asymmetry; CVAI, cranial vault asymmetry index.

Figure 4 shows the measurement method using CA and CVAI. CA (mm) was defined as the difference between the two diagonal cranial diameters, 30° from the Y-axis and calculated as follows: CA = diagonal A − diagonal B. CVAI was calculated as follows: CVAI (%) = CA/diagonal B × 100% (where diagonal A is greater than B) [9,15]. Head circumference was defined as the circumference of the measurement plane.

### 2.7. CHT

CHT is a treatment in which an infant wears a custom-made helmet to bring the head to a normal shape (Aimet^®^, Japan Medical Company Inc. Tokyo, Japan; medical device approval number: 30100BZX00022000). CHT was introduced at the request of parents or guardians. The applied protocol was as follows: the infants wore the helmet for 23 h a day after setting a break-in period of 7–14 days to increase the wearing period gradually. The infants visited our medical center within 3 to 4 weeks after the therapy start to check the fit of the helmet. If the infants developed any side effects, such as skin injury, by wearing the helmet, the parents or guardians were instructed to visit our medical centers with the infants.

### 2.8. Statistical Analyses

Prior to commencing this study, no confirmatory statistical tests were performed due to the lack of previous studies employing similar methodology; therefore, power calculation could not be performed. Statistical analyses were performed using the non-parametric Mann–Whitney U-test, chi-square test, and multiple logistic regression analysis. The median (minimum–maximum) values and number (percentage) are presented in the results. Statistical analyses were performed using JMP software (version 14; SAS Institute Inc., Tokyo, Japan). Statistical significance was set at *p* < 0.05.

## 3. Results

### 3.1. Study 1

#### 3.1.1. Clinical Characteristics and Measured Values

Of the 56 infants with severe plagiocephaly (i.e., natural course), 37 (66%) remained severe (no-change group) and 19 (34%) improved to moderate or mild (improved group). Table 1 displays a comparison between the no-change and improved groups following the natural course. No statistically significant differences were found in the evaluation age, evaluation interval, delivery mode, fetal presentation, and nutrition. In the no-change group, the median CA at initial evaluation (median age, 3 months) was 15.6 mm, and in the second evaluation (median age, 6 months), CA subsided by −0.2 mm (median, 14.9 mm). In the improved group, the median CA at initial evaluation (median age, 3 months) was 13.5 mm, and in the second evaluation (median age, 6 months), CA subsided by −2.8 mm (median, 11.0 mm). No significant differences were found in initial and second head circumference and growth of head circumference between the groups.

#### 3.1.2. Natural Course of Severe Plagiocephaly

**Figure 5:**
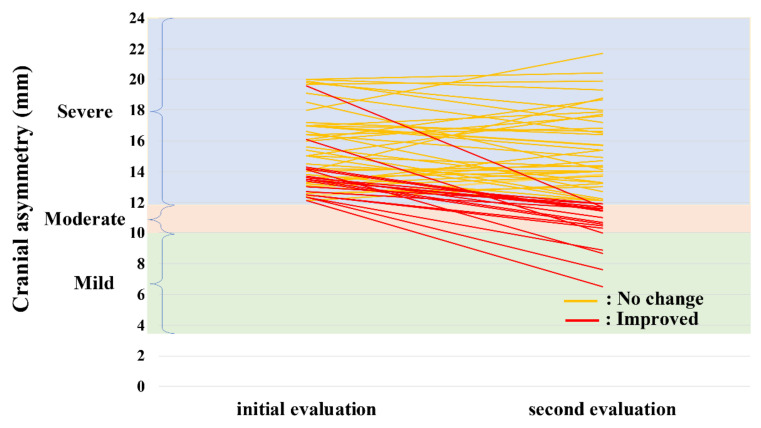
Initial and second evaluation CA values of infants with plagiocephaly in the improved and no-change group. CA, cranial asymmetry.

Figure 5 shows the CAs of the initial and second evaluations in the improved and no-change groups of infants with severe plagiocephaly. Of the 56 enrolled infants, 37 (66%) did not improve, with no change from “severe”. The severity of the improved group transitioned to “moderate” in 14 and “mild” in five infants. The no-change group had an initial CA of 15.6 mm, whereas the improved group had an initial CA of 13.5 mm (*p* = 0.001), suggesting that a higher CA value was associated with severity in the second evaluation.

### 3.2. Study 2

#### 3.2.1. Clinical Characteristics and Measured Values

Table 2 shows comparisons of obstetric factors and evaluation age between the natural-course (*n* = 24) and CHT (*n* = 33) groups. As expected, there was no significant difference in evaluation age since it was matched (*p* = 0.874). The evaluation interval between initial and second evaluation ages was similar in the two groups (*p* = 0.564). There was a significant difference in sex only between the two groups (male-sex prevalence was 83% and 57% in the natural-course and CHT groups, respectively, *p* = 0.039). No statistically significant differences were found in the delivery mode, fetal presentation, and nutrition.

Table 3 shows comparisons of CA and CVAI between the natural-course and CHT group. The median CA at first evaluation (median age, 4 months) was 14.6 mm in the natural-course group and 15.0 mm in the CHT group (*p* = 0.348). The CA was reduced by −1.6 mm in the natural course group (median 14.1 mm) and −4.6 mm in the CHT group (median, 11.6 mm) (*p* < 0.001). No significant differences were found in initial and second head circumference and growth of head circumference between the groups (Table 3).

#### 3.2.2. Efficacy of CHT in Severe Plagiocephaly

**Figure 6:**
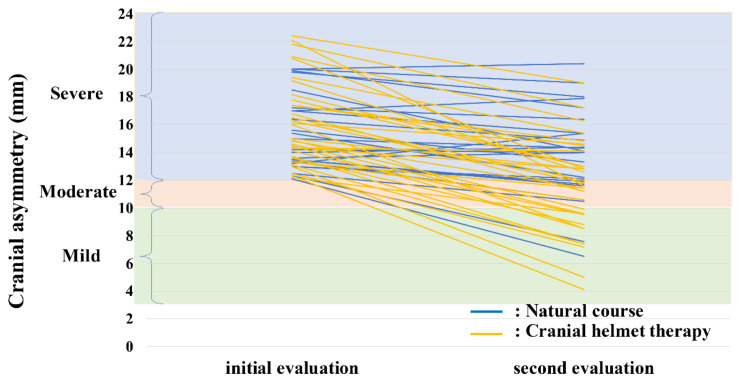
Initial and second evaluation CA values of infants with plagiocephaly in the natural-course and CHT groups. CHT, cranial helmet therapy.

Figure 6 displays the CAs of the initial and second evaluations in the natural-course and CHT groups of infants with severe plagiocephaly. Seven of the 24 infants in the natural-course group improved (29%; 5 moderate and 2 mild), whereas 19 of 33 the infants in the CHT group improved (58%; 7 moderate and 12 mild), with a significantly higher rate in the CHT than in the natural-course group (*p* = 0.034). The improvement in CA value in the CHT group was approximately three times that of the natural-course group (Table 3). Multiple logistic regression analysis was performed using sex and CA-improvement value that were significantly different between the natural-course and CHT groups by the univariate analysis. A CA-improvement value was identified as being independently associated with the CHT (odds ratio 0.45, 95% confidence interval 0.28–0.65; *p* < 0.001, Table 4). In the CHT group, no head circumference growth disorder was not observed. Additionally, there were no skin injuries that discontinued CHT.

## 4. Discussion

This study had two novel findings. First, 66% of infants with severe plagiocephaly following the natural course did not demonstrate any improvement. Obstetric factors and nutritional status were not associated with worsening. Second, even in the short study period, approximately 60% of the infants with severe plagiocephaly who received CHT exhibited improvement, an improvement that was approximately twice that in the natural course. Furthermore, the improvement in CA value due to CHT was three times that in the natural course. The improvement efficacy by CHT was consistent with that of other previous studies [8,9,10,11,12], although the study design and evaluation methods were different. Our current study and other studies have shown that the degree of improvement by CHT was significantly greater than the natural course and repositioning or physiotherapy only [9,12].

In our clinical practice, we already noticed that many infants with severe plagiocephaly who followed the natural course did not show any improvement at their health checkups. This study revealed that approximately 70% of severe plagiocephaly did not improve. The percentage of infants with severe plagiocephaly who improved to mild (i.e., no indication for CHT) was only 7%. Therefore, we recommend that pediatricians caution parents regarding the unlikely natural improvement associated infants with severe plagiocephaly with CA > 12 mm. As our results revealed that plagiocephaly with a median CA of 15.6 mm or more is likely to remain “severe” after 2–3 months, an infant with a higher CA among those with CA > 12 mm at the initial evaluation may indicate the need for CHT.

We also found that the improvement in CA value in the CHT group was approximately three times higher than that in the natural-course group. Many reports have proven the efficacy of CHT in the treatment of severe plagiocephaly, with a recommendation to initiate CHT at 4–8 months of age [2,6,12,13,14]. Therefore, we conducted an age-adjusted matched case-control study of 4–8 months. No previous studies have conducted a comparison between the natural-course and CHT outcomes among infants with plagiocephaly of the same severity and evaluation age. While several reports have shown differences in the evaluation period or severity of initial asymmetry between the CHT and natural-course groups [11,12,16], our study showed no difference in the evaluation interval period, evaluation age, and severity of initial asymmetry between the two groups. Male infants are generally a risk of severe plagiocephaly [6]. In this study, a significant reduction in CA was found in infants with severe plagiocephaly who underwent CHT by Uni- and multi-variate statistical analyses. Therefore, infants with severe plagiocephaly who received CHT achieved a considerably superior outcome, even within a short period. The effect of CHT as a treatment for infants has been reported to improve within 2–3 months, as noted in other studies [17,18], indicating that our results were in agreement with those of these studies [17,18]. Even in a median evaluation interval of two months, our study showed that CHT for severe plagiocephaly is superior to the natural course. However, because it is still unknown whether the two groups will improve or worsen over a longer period, further studies will be required on this topic.

We focused on CA in this study; nevertheless, the severity is defined using CVAI in other previous studies [3,15,19,20] (e.g., mild, 5–6%; moderate, 7–9%; severe, 10–13%; and very severe, >14%) [3,15]. The median CVAI exceeded 10% in both the natural-course and CHT groups in this study 2, indicating a severe plagiocephaly.

Our study has certain limitations. First, the evaluation interval period was brief. In a randomized controlled trial for CHT, the outcomes were evaluated at 24 months of age [21,22]. Currently, we did not have the long follow-up data over 12 to 24 months of age. Our next study with an endpoint at 1.5 years of age is currently underway. However, the growth potential of the head, as the most important factor for improvement, predominates within the first year of life. Therefore, this study aimed to clarify the natural course of severe plagiocephaly without CHT within the first year of life. The second was the confounding effect of supportive interventions, such as repositioning. Unfortunately, we did not collect the data regarding supportive interventions. The intensity, duration, and frequency of the applied specific methods varied slightly between observed infants. These are confounding factors and may affect the outcome. Further prospective clinical studies on the effects of CHT considering supportive interventions are needed. However, in this study, because parents were asked to continue any previously initiated further supportive interventions, we believe that all infants underwent similar supportive interventions, regardless of whether they had CHT. Finally, as side effects of CHT, only skin injury and head circumference growth of the infants were checked. Others, such as a quality of life or psychomotor development of the infants with CHT and satisfaction and anxiety of the parents, should be investigated in the future study.

## 5. Conclusions

Severe plagiocephaly with CA > 12 mm did not improve in 66% of infants without CHT. An infant with a higher CA among those with CA > 12 mm at the initial evaluation may be an indication for CHT consideration. Approximately 60% of infants with severe plagiocephaly who received CHT improved, and the improvement value of CA was three times that in the natural course.

## Figures and Tables

**Table 1:** Comparisons between the no-change and improved groups in the natural course of severe plagiocephaly.

	No-Change Group *n* = 37	Improved Group *n* = 19	*p*-Value
Birth weight, g	3070 (1962–4144)	3100 (1886–3820)	0.729
Birth order (first born)	23 (62)	11 (58)	0.757
Sex (male)	23 (62)	12 (63)	0.942
Gestational age at birth, weeks	39 (35–41)	38 (34–41)	0.199
Initial evaluation age, months	3 (1–10)	3 (1–10)	0.634
Second evaluation age, months	6 (3–14)	6 (3–12)	0.328
Evaluation interval, months	2 (1–7)	2 (1–4)	0.221
Initial evaluation CA, mm	15.6 (12.1–20.0)	13.5 (12.1–19.6)	0.001
Second evaluation CA, mm	14.9 (12.1–21.7)	11.0 (6.5–11.9)	<0.001
CA-improvement value, mm	−0.2 (−4.4–4.8)	−2.8 (−7.9–−1.1)	<0.001
Initial evaluation CVAI, %	10.7 (8.0–14.4)	9.4 (7.9–13.2)	0.010
Second evaluation CVAI, %	9.9 (7.8–14.7)	7.6 (4.3–8.5)	<0.001
CVAI-mprovement value, %	−0.5 (−3.1–2.3)	−2.1 (−5.6–−0.9)	<0.001
Initial head circumference, mm	418.2 (351.3–472.3)	410.1 (380.3–460.7)	0.222
Second head circumference, mm	441.6 (393.4–496.7)	432.6 (406.4–468.2)	0.253
Growth of head circumference, mm	16.9 (6.5–42.1)	17.7 (7.5–36.2)	0.876
Delivery mode			
Vaginal delivery	22 (60)	11 (58)	0.746
Cesarean section	11 (30)	7 (37)	
Vacuum delivery	2 (5)	1 (5)	
Forceps delivery	2 (5)	0 (0)	
Fetal presentation			0.696
Cephalic presentation	34 (92)	18 (95)	
Breech presentation	3 (8)	1 (5)	
Nutrition			0.067
Breastfeeding	4 (11)	7 (37)	
Formula feeding	6 (16)	2 (10)	
Mixed feeding	27 (73)	10 (53)	

Values are shown as median (range) or number (percentage). CA, cranial asymmetry; CVAI, cranial vault asymmetry index.

**Table 2:** Comparisons of obstetric factors and evaluation age between the natural-course and cranial helmet therapy groups.

	Natural-Course Group *n* = 24	CHT Group *n* = 33	*p*-Value
Birth weight, g	3148 (1886–4144)	3130 (2512–3642)	0.651
Birth order (first born)	16 (67)	18 (55)	0.357
Sex (male)	20 (83)	19 (57)	0.039
Gestational age at birth, weeks	40 (34–41)	39 (37–41)	0.194
Initial evaluation age, months	4 (4–8)	4 (4–8)	0.874
Second evaluation age, months	6.5 (5–14)	7 (6–10)	0.822
Evaluation interval, months	2 (1–7)	2 (1–4)	0.564
Delivery mode			0.804
Vaginal delivery	17 (71)	20 (60)	
Cesarean section	5 (21)	9 (27)	
Vacuum delivery	1 (4)	3 (10)	
Forceps delivery	1 (4)	1 (3)	
Fetal presentation			0.316
Cephalic presentation	24 (100)	30 (91)	
Breech presentation	0 (0)	2 (6)	
Transverse presentation	0 (0)	1 (3)	
Nutrition			0.253
Breast feeding	6 (25)	12 (36)	
Formula feeding	7 (29)	4 (12)	
Mixed feeding	11 (46)	17 (52)	

Values are shown as median (range) or number (percentage). CHT, cranial helmet therapy.

**Table 3:** Comparisons of CA and CVAI between the natural-course and cranial helmet therapy groups.

	Natural-Course Group *n* = 24	CHT Group *n* = 33	*p*-Value
Initial evaluation CA, mm	14.6 (12.1–20.0)	15.0 (12.2–22.4)	0.348
Second evaluation CA, mm	14.1 (6.5–20.4)	11.6 (4.1–19)	0.011
CA-improvement value, mm	−1.6 (−5.6–2.2)	−4.6 (−10.5–0.2)	<0.001
Initial evaluation CVAI, %	10.0 (8.0–13.2)	10.3 (8.1–14.9)	0.316
Second evaluation CVAI, %	9.0 (4.3–12.5)	7.8 (2.8–12.2)	0.023
CVAI-improvement value, %	−1.43 (−3.9–1.2)	−3.3 (−7.1– −0.3)	<0.001
Initial head circumference, mm	440.9 (395.0–466.2)	432.6 (411.2–456.7)	0.365
Second head circumference, mm	454.1 (412.2–496.7)	446.2 (425.7–469.7)	0.157
Growth of head circumference, mm	15.0 (6.5–33.0)	12.9 (1.4–24.2)	0.121

Values are shown as median (range). CA, cranial asymmetry; CHT, cranial helmet therapy; CVAI, cranial vault asymmetry index.

**Table 4:** Multivariate statistical analysis.

	Odds Ratio (95% Confidence Interval)	*p*-Value
Sex (female/male)	3.52 (0.80–18.6)	0.0981
CA-improvement value	0.45 (0.28–0.65)	<0.001

CA, cranial asymmetry.

## Data Availability

The data presented in this study are available on request from the corresponding author.
